# Project SWITCH Study Protocol: A Tobacco-Free Workplace Program for Dissemination and Implementation in Lung Cancer Screening Centers

**DOI:** 10.3390/mps8040070

**Published:** 2025-07-01

**Authors:** Ammar D. Siddiqi, Maggie Britton, Isabel Martinez Leal, Matthew Taing, Tzuan A. Chen, Lisa M. Lowenstein, Jennifer A. Minnix, Lorraine R. Reitzel

**Affiliations:** 1 Department of Behavioral Science, University of Texas MD Anderson Cancer Center, 1515 Holcombe Blvd., Houston, TX 77030, USA; 2 Department of Management, Policy, and Community Health, School of Public Health, University of Texas Health Science Center at Houston, 1200 Pressler St., Houston, TX 77030, USA; 3 Dell Medical School, University of Texas at Austin, 1501 Red River St., Austin, TX 78712, USA; 4 Department of Psychological, Health, and Learning Sciences, University of Houston, 491 Farish Hall, Houston, TX 77204, USA; 5 HEALTH Research Institute, University of Houston, 4349 Martin Luther King Blvd., Houston, TX 77204, USA; 6 Department of Health Services Research, University of Texas MD Anderson Cancer Center, 1155 Pressler St., Houston, TX 77030, USA

**Keywords:** tobacco, smoking, lung cancer, intervention, tobacco-free workplace policy, tobacco cessation, Texas, cancer prevention, implementation science, mixed methods

## Abstract

Background/Objectives: Cigarette smoking has been causally linked to 90% of all cases of lung cancer, contributing to its high mortality rate. Lung cancer screening centers offer low-dose computed tomography, the only recommended diagnostic screening tool for lung cancer detection. A previous Texas-based study found that centers with lung cancer screening programs failed to consistently provide evidence-based tobacco cessation and relapse prevention interventions recommended by clinical practice guidelines to their patients, who are primarily people who currently or previously smoked. This represents a missed opportunity to assist patients by providing evidence-based tobacco use care during a particularly relevant clinical encounter. Methods: To improve cigarette smoking cessation care delivery and relapse prevention in this setting, this protocol paper seeks to provide a framework for adapting Taking Texas Tobacco Free, a comprehensive, evidence-based tobacco-free workplace program, to lung cancer screening centers. The adapted program, Project SWITCH, will be developed through a formative evaluation process with center stakeholders to identify proactive adaptations to programming based on center-specific contexts. Project SWITCH is expected to be implemented in at least nine lung cancer screening centers in Texas and will be disseminated more broadly to centers statewide. Results: Quantitative and qualitative data will be collected from multiple stakeholders throughout the intervention using a convergent parallel mixed methods design to make additional program adaptations and comprehensively evaluate the achievement of the project’s implementation and dissemination goals. Conclusions: Results from this project’s implementation and dissemination phases are expected to reduce lung cancer morbidity and mortality in Texas by providing an evidence-based, sustainable framework for tobacco-free workplace programs in this specific setting that improves cancer prevention and control practices.

## 1. Introduction

The use of tobacco products, including cigarettes and non-cigarette tobacco products, is detrimental to human health and can contribute to the development of lung disease, heart disease, and cancers, among other diseases [1,2]. Cigarette smoking, the most common form of tobacco use in the United States (US), has notably been causally linked to over 12 different types of cancers [3], 20% of all cancer diagnoses [4], and 30% of all cancer deaths, contributing to its designation as the leading preventable cause of cancer in the US [5]. Despite improvements in prevention and treatment, cancer remains the second leading cause of death [6]. In particular, lung cancer is the deadliest cancer, accounting for 20% of the more than 600,000 cancer-related deaths annually in the US [7]. The elevated mortality from lung cancer can specifically be attributed to the strong causal link with cigarette smoking and cigarette smoke exposure [8]. Nearly 90% of lung cancer diagnoses and deaths are caused by cigarette use, with smoking increasing one’s risk of developing lung cancer by 15- to 30-fold. Consequently, improving lung cancer prevention is paramount in reducing the sheer morbidity and mortality associated with cancer.

Low-dose computed tomography (LDCT) is the only recommended screening test for lung cancer [9,10]. Lung cancer screening centers (or functionally equivalent facilities) offer LDCT to people at high risk for developing lung cancer. A high-risk person is defined by the US Preventive Services Task Force (USPSTF) as someone who, among other requirements, is 50 to 80 years old, has a 20-pack-year cigarette smoking history, and who either currently smokes cigarettes or has quit smoking cigarettes in the past 15 years [11]. Thus, lung cancer screening centers are ideal for delivering evidence-based tobacco control interventions, especially for patients who smoke cigarettes and are diagnosed with lung cancer, as quitting at the time of diagnosis markedly improves survival [12].

In recent years, the landscape of lung cancer screening in the US has changed, making the delivery of evidence-based tobacco control interventions within lung cancer screening centers especially timely. In 2021, the USPSTF updated its 2013 recommendation to expand lung cancer screening by lowering the starting age (from 55 to 50) and the smoking history requirements (from 30 to 20 pack-years) [11,13]. In 2022, the Centers for Medicare & Medicaid Services (CMS) followed suit and revised their decision on reimbursement for beneficiaries to align with the USPSTF recommendations [14]. These changes have significantly increased the number of people eligible for, and who can receive reimbursement for, lung cancer screening with LDCT from 8 million to 14.5 million [15], with particular implications for Black adults in the US [16], who tend to smoke later in life and with lower intensity but who bear a disproportionate burden of lung cancer incidence and death [17] and are more likely to be diagnosed younger [18].

Approximately half of patients eligible for lung cancer screening currently use cigarettes, and half previously used cigarettes, underscoring the need for lung cancer screening centers to deliver tobacco cessation care and support relapse prevention for their patients [19]. Unfortunately, lung cancer screening centers have struggled to provide tobacco cessation care and support relapse prevention [20], despite cigarette dependence being characterized as a chronic, relapsing disease [21,22]. In 2022, a study that included 63 lung cancer screening centers across Texas, US, reported that they have generally been unable to furnish active, evidence-based smoking cessation interventions [20]. Specifically, about 10% of centers never asked or asked only a proportion of their patients about whether they currently smoke cigarettes, one-third did not always advise people who smoke to quit, and nearly 70% never assessed patients’ readiness to quit. Although 68% of lung cancer screening centers provided self-help smoking cessation material, 81% never provided cessation counseling, 85% never recommended cessation medications, and 68% and 77% never referred people who smoked to onsite cessation services or quitlines, respectively. Unfortunately, many of these practices are contrary to clinical practice guidelines, which recommend that providers consistently deliver interventions and facilitate the use of cessation medications and services [23].

Prior to 2022, lung cancer screening centers were required by CMS to provide smoking cessation interventions for LDCT reimbursement purposes. However, to eliminate barriers to the lung cancer screening process, since 2022, the CMS no longer requires Medicare beneficiaries to receive cessation interventions in LDCT centers, though it still acknowledges their importance [14]. Nonetheless, to be registered as a center of excellence (COE) through the GO2 Foundation for Lung Cancer, centers must either include a comprehensive tobacco cessation program with screening for people who currently smoke or refer them to one (among other criteria) [24]. Among the nearly 800 COE centers nationwide, only 16 (<2%) are in Texas, US [25]. With at least 94 lung cancer screening centers across Texas, there is substantial room for improvement in this metric [26]. Thus, there is support (from major medical organizations), motivation (for centers to receive the coveted COE designation), and opportunity (only ~17% of centers in Texas are designated as a COE) for lung cancer screening centers to provide evidence-based tobacco use interventions, as well as a need for capacity-building efforts in this setting.

Several barriers at the patient, provider, and center/organizational level may contribute to the limited tobacco cessation care provision in lung cancer screening centers [27,28]. In this setting, patients occasionally refuse tobacco use screenings or participation in cessation programs due to difficulty managing stress, low motivation, and poor social support [28,29]. However, this is the minority of patients, particularly consisting of those who often have limited knowledge of smoking-related health effects [28], as the vast majority of people who smoke (~68%) endorse an interest in quitting [30]. Barriers also exist at the provider level, where limited knowledge about tobacco cessation and a lack of time and reimbursement have been reported to subvert cessation care delivery [27,28]. This is despite the significant impact of tobacco dependence on lung cancer screening patients’ health outcomes [12], the existence of brief intervention and referral options [23], and most insurance providers being mandated to provide coverage for tobacco cessation treatment [31]. At a center/organizational level, leadership and personnel may not prioritize the treatment of tobacco dependence, which can influence organizational culture and limit resource provision for tobacco cessation [28]. Consequently, there is a translational gap in need of remedy whereby evidence-based interventions to curtail cigarette smoking are not being offered to patients in accordance with guidelines that recommend all healthcare providers address tobacco use at every clinical contact [23].

Tobacco-free workplace programs that include the adoption of a comprehensive tobacco-free workplace policy (no tobacco use/vaping anywhere on site), staff education/training about the harms of and how to treat tobacco use, and resource provision (e.g., where and how to receive care) are evidence-based interventions to build organizational capacity to address patients’ (and staffs’) tobacco use and to prevent relapse. Since 2013, the research team has been implementing and disseminating a tobacco-free workplace program to reduce tobacco use health risks among patients and staff in a variety of healthcare and community settings across Texas, US, including centers serving people experiencing homelessness [32] and clinics serving people who have behavioral health needs (substance use disorders and/or mental health diagnosis) [33,34,35]. According to social cognitive theory, programming components can drive behavioral changes in diverse settings by targeting environmental and personal factors and behavioral capability [36]. Through processes such as observational learning and reciprocal determinism, individuals adapt their behaviors to their social context. Following program implementation, changes in tobacco cessation capacity have included greater tobacco-related knowledge and training among staff [35,37,38], more consistent screening and intervention delivery by care providers [32,34,39], and provision of cessation medication and counseling to patients and staff [32,33,40]. Each of these respective components is evidence-based and adapted to each setting using implementation strategies suited to the context and an iterative mixed methods evaluation process.

Tobacco-free workplace programs have the potential to prevent lung cancers among at-risk adults and to improve health outcomes associated with lung cancer diagnosis when implemented in lung cancer screening centers. The objective of this protocol paper is to provide a detailed overview of Project SWITCH (Supporting Workplace Investment in Tobacco Control and Health), a tobacco-free workplace program that will be adapted for implementation within lung cancer screening centers across Texas, US (promoted broadly for self-led dissemination within Texas and the US). Project SWITCH is an implementation study project that seeks to understand how to bridge the gap between what we know (evidence) and what we do (practice), ultimately improving population health outcomes. This protocol paper will discuss the process of program adaptation to participating lung cancer screening centers, plans for implementation, and plans for assessing both implementation and intervention outcomes. Brief contextual information about the centers participating in the protocol and where they are in the implementation process as of the time this paper was written will also be provided. It will also review the methods and objectives associated with the dissemination phase of the initiative. Project SWITCH is expected to provide a framework for improving tobacco-dependence care in lung cancer screening centers to reduce cancer incidence and mortality by facilitating compliance with public health guidelines [23].

## 2. Materials and Methods

### 2.1. Institutional Approvals and Funding

Project SWITCH is focused on quality improvement in healthcare practice; it does not meet the regulatory definition of human subjects research. In anticipation of funding by the Cancer Prevention and Research Institute of Texas, the study procedures were approved by the Quality Improvement Assessment Board at the University of Texas MD Anderson Cancer Center on 2 February 2023. Funding for this dissemination and implementation study was obtained on 1 March 2023 under prevention award #PP230002; the current grant contract end date is 28 February 2026.

### 2.2. The Intervention’s Core Components

**Figure 1:**
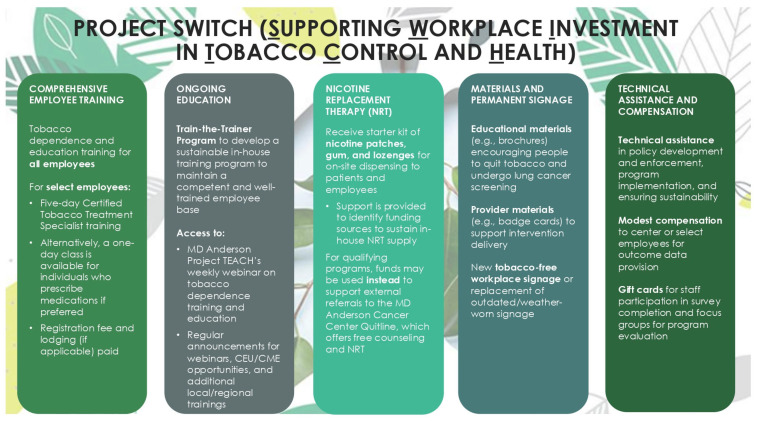
Various components of Project SWITCH, a tobacco-free workplace program for lung cancer screening centers in Texas, US (2023–2026).

The tobacco-free workplace program that has been implemented in other healthcare and community settings over the last decade in Texas, US, will form the basis of our approach. As such, Project SWITCH has the following evidence-based core program components: (1) the adoption and rollout of a comprehensive tobacco-free workplace policy, (2) staff and provider education about tobacco use and how to quit, inclusive of the development of a sustainable in-house tobacco education program, and (3) the integration of tobacco use assessments into regular patient care and the provision of evidence-based cessation services (including referrals) for patients and staff who use tobacco and want to quit. Figure 1 illustrates how these core components are presented to potential centers during the recruitment process, which is discussed in more detail in the following sections. Specifically, they are framed around training and skill development opportunities, as well as the provision of policy and resource support.

### 2.3. Participatory Approach to Adaptation and Implementation

The study team anticipates the need for adaptations to both the materials and the methods of the existing tobacco-free workplace program. This process will be broadly guided by the Exploration, Preparation, Implementation, Sustainment (EPIS) framework [41,42], with the goal of highlighting common and unique elements of lung cancer screening centers relative to prior program partners, inclusive of their inner and outer contexts and the factors that connect the two.

Modifications to the intervention and implementation strategies will include proactive adaptations to fit the local context gathered from different key stakeholders during the formative phase of the work. Proactive adaptations accord with the Planned Adaptation approach that provides a guide on how adaptations affect fidelity (i.e., align with the logic model and content of the program) [43]. While reactive adjustments occurring during the active implementation process will also be included, researchers will work with program partners to ensure their refinement aligns with the program goals and theory. In all cases, adaptations will be documented using the Framework for Reporting Adaptations and Modifications-Enhanced (FRAME) and the Framework for Reporting Adaptations and Modifications to Evidence-based Implementation Strategies (FRAMES-IS) [44,45].

#### 2.3.1. Proactive Modifications

The approach to proactive modifications will be systematically guided by the steps outlined by Escoffery and Miller and colleagues, which are primarily based on stakeholder consultation [44,46]. The study team will first review the extant literature, noting factors that may need to be considered for adaptation and that will inform data collection in the exploration phase. Then, information will be gathered through quantitative and qualitative formative evaluation procedures with stakeholders (e.g., leadership, staff, and patients) from participating lung cancer screening centers to further inform potential adaptations to the local context. The proactive adaptation process for materials and methods will consider the unique setting dynamics of each participating center, including its workflow, facilitators, and barriers. The study team will meet weekly to discuss findings from these sources as potential proactive modifications are considered.

Each lung cancer screening center that enrolls in Project SWITCH will also have one or more “program champions,” employees who are appointed by their leadership to work closely with the study team to actively implement the program in their setting. It will be advised that program champions have the capacity to facilitate program adoption and possess strong skills in organization, coordination, and planning. The program champion will actively partner with the study team implementor (typically a Health Education Specialist) to further inform proactive adaptations in a collaborative, participatory context and ensure their feasibility for participating centers. The study team will discuss the results of these formative communications weekly to ensure that the planned adaptations are in keeping with the underlying evidence base of the intervention’s core components (i.e., if they are “fidelity consistent/core elements preserved” or “fidelity inconsistent/core elements changed”) [44].

#### 2.3.2. Reactive Modifications

During the active implementation, the study team implementer will meet with the site program champion to discuss the implementation and understand any on-the-ground challenges and any center-led changes to the implementation process that may have been made without prior consultation with the study team. Any center-specific reactive modifications will be discussed further with the study team in an iterative process to ensure fidelity with the evidence base and appropriate documentation for later evaluation. When adaptations are fidelity inconsistent, the study implementer will work closely with the program champion/s to inform them of concerns and provide guidance on alternative approaches. However, if the center continues to insist on an inadvisable approach, the study team will document the situation and adaptation accordingly.

### 2.4. Goals and Objectives

Project SWITCH has three goals, each with up to three objectives. The three overarching goals are as follows: (1) adapt our evidence-based tobacco-free workplace intervention to a new setting: lung cancer screening centers; (2) implement the program in nine or more lung cancer screening center locations; and (3) disseminate the adapted program statewide via a step-by-step implementation guide, resource provision, and technical assistance (i.e., guidance) for center-led implementation.

#### 2.4.1. Goal 1 Objectives: Adaptation of Materials and Methods

The objectives associated with Goal 1 are to create/tailor the following: (1) educational materials for program implementation to include a Project SWITCH training curriculum for live and recorded training provision, and (2) health education materials for patients in English and Spanish (and possibly other languages according to populations served) and reference materials for healthcare providers in English. Together, the formative evaluation and information gathered during active program implementation will inform the final objective: the creation of (3) a step-by-step implementation guide for Project SWITCH’s implementation in lung cancer screening centers.

#### 2.4.2. Goal 2 Objectives: Implementation

Project SWITCH will use a Hybrid Type 3 mixed methods design [47], which consists of testing an intervention implementation and primarily collecting data on the implementation outcomes, but also studying those related to effectiveness. Therefore, the primary outcomes of interest in Goal 2 are related to implementation; intervention/effectiveness outcomes (e.g., smoking abstinence) are gathered but are secondary outcomes. This is because the workplace intervention is already evidence-based; thus, the implementation outcomes are of interest as key indicators of implementation success and intermediate objectives on the path to intervention/effectiveness outcomes.

Implementation objectives were identified using Proctor’s Implementation Outcomes framework [48]; they are (1) the penetration of education/knowledge to staff within partner centers; (2) the fidelity to (a) public health guidelines/best practices in the delivery of tobacco use care to patients, and (b) Project SWITCH’s core components as reflected by program completion; and (3) the sustainment of tobacco use care throughout a 6-month period post-Project SWITCH’s implementation.

**Figure 2:**
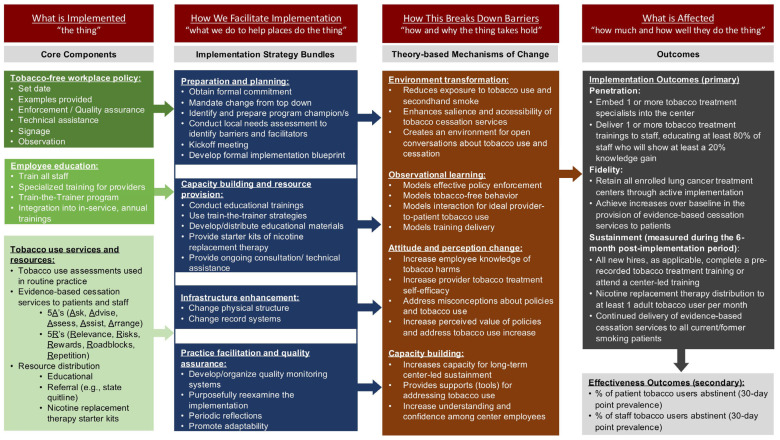
Core implementation components, strategies, and outcomes for Project SWITCH, a tobacco-free workplace program for lung cancer screening centers in Texas, US (2023–2026). Some terminology is informed by Curran (2020) and Powell (2015) [49,50].

Table 1 includes the implementation objectives and their operationalization. Their measurement is additionally detailed in a subsequent section (see Section 2.5.4. Data Collection from Participating Centers). Figure 2 displays the core implementation components, implementation strategies, and implementation and intervention objectives for Project SWITCH.

#### 2.4.3. Goal 3 Objectives: Dissemination

The final objectives are to disseminate Project SWITCH to lung cancer screening centers statewide (including in rural and medically underserved areas of the state) and provide at least two centers with technical assistance for self-led implementation. To achieve this, we will create (1) a dedicated webpage to disseminate the adapted program materials (e.g., health education materials and provider trainings). We will also (2) passively and actively disseminate the tailored program and materials to (a) lung cancer screening centers not participating in adaptation/implementation (Goals 1–2), (b) the community at large, and (c) academic audiences. Further, we will (3) provide consultation and hands-on technical assistance to lung cancer screening centers not involved in Goals 1–2 that are interested in self-led program implementation.

### 2.5. Study Design

#### 2.5.1. Center Recruitment and Enrollment

The inclusion criteria for Project SWITCH are as follows: (1) a lung cancer screening center (or program) located in Texas, with (2) leadership interested in participation and willing to work with us to implement the program’s components and provide data to guide and evaluate implementation. The focus on Texas is due to funder requirements: the Cancer Prevention and Research Institute of Texas was voted into fruition by Texans in 2007 and renewed to continue in 2019 to address the burden of cancers in the state. The Cancer Prevention and Research Institute of Texas funds prevention work, including implementation science studies like Project SWITCH. The Cancer Prevention and Research Institute of Texas is the largest state-level cancer research investment in the US and the second-largest cancer research and prevention program globally. It is a National Cancer Institute-certified funder based on its rigorous grant application peer review process.

Originally, we were to implement the program within nine lung cancer treatment centers that were part of a collaborator’s healthcare system across Harris County, Texas. The collaborator’s effort would be supported via a subcontract. However, between the grant application and funding, the collaborator moved out of state, requiring the engagement of new centers. The location and contact information of lung cancer screening center locations in Texas, US, will be identified using a contact list from a prior study [20] and via internet searches. Recruitment of lung cancer screening centers is achieved through emails to the centers about the program, attendance as exhibitors to relevant meetings to spark interest (e.g., 2023 Innovations in Cancer Prevention and Research Conference and Texas Radiological Society Conference), and via presentations to various stakeholders and workgroups (e.g., Cancer Alliance of Texas’ lung cancer screening priority area workgroup).

Enrollment for Project SWITCH is at the level of the center. Interested parties identified through recruitment strategies will meet with a study team representative, virtually or in person, who will lead a short presentation about the program and answer any questions. Enrollment in Project SWITCH is at the lung cancer screening center leadership representative’s discretion and formalized through a signed Memorandum of Understanding.

#### 2.5.2. Study Participants

Study participants for Goals 1 and 2 comprise all enrolled center staff, including leadership, direct service providers, and employees who are not direct care providers. The study team will work with each center’s leadership to identify one or more employees at each center who will serve as the program champion and the liaison to the study team. Study participants are engaged in quantitative data collection and (for a subset) qualitative data collection. Study participants for Goals 1 and 2 can also include center patients, who can engage in qualitative data collection procedures with the permission of leadership. Because this is a real-world quality improvement program, the exact number of study participants will be unique to the centers that participate in the program.

There will be no individual participants for Goal 3, the dissemination arm of this project, from whom data are collected. The study team works with the appropriate designee of lung cancer screening centers (that did not participate in Goals 1 and 2) to fill resource requests (e.g., printed educational materials) and to provide technical assistance (e.g., policy implementation). The study team tracks Goal 3 data (e.g., the number of printed materials ordered and number of technical assistance sessions) using program records (e.g., spreadsheets).

#### 2.5.3. Implementation Process for Participating Centers

Project SWITCH’s implementation rollout (Goal 2), guided by the EPIS framework, will be ongoing within respective lung cancer screening centers following their enrollment. Depending on the center and its capacity, the active implementation phase can take 12 months or more. During implementation, we will simultaneously tailor materials to the setting/s using empirical data and stakeholder feedback (Goal 1). Project SWITCH’s implementation process components (including implementation strategies), process objectives, and alignment with EPIS phases [primary (secondary)] are in Table 2. Our team will work closely with the center program champion and staff through site visits and weekly correspondence/meetings throughout implementation for quality assurance.

#### 2.5.4. Data Collection from Participating Centers

Data collection is designed to assess both the implementation of Project SWITCH by capturing components of the underlying social cognitive theory (e.g., behavioral, environmental, and social factors), EPIS framework determinants (e.g., center characteristics for inner context), and the planned program evaluation. To achieve this, quantitative and qualitative data will be collected. Various data sources are described below and largely pertain to the active implementation objectives (Goal 2) unless otherwise specified. Although Project SWITCH is considered a quality improvement study and does not meet the regulatory definition of human subjects research, all quantitative and qualitative procedures are guided by best practices, inclusive of an informed consent process that outlines how data provided will be used and maintained and procedures related to confidentiality and privacy.

*Study team records.* The Project SWITCH team will track implementation (Goal 2) and dissemination (Goal 3) progress using an internal spreadsheet. This sheet will include, for example, information about resources distributed, staff training completion, the number of technical assistance/consultations provided, and the number of centers reached via email.

*Pre- and post-implementation surveys*. Center leadership will take a pre-implementation survey to assess center characteristics and readiness for program adoption and sustainment. This survey will be prefaced by a cover letter with elements of informed consent and accessed by a member of the center leadership team via a Qualtrics link. The leadership survey includes face-valid, investigator-generated questions about the center, its staff, and its patients. Through the generated results, study staff will learn more about the center and its stakeholders, which can inform implementation rollout (Goal 2) as well as guide material tailoring (Goal 1). Remuneration offered for the completion of this survey is $40 per survey/center in the form of a department store gift card.

General employees and direct service providers will be requested to complete a separate survey at pre- and post-implementation, with program champions assisting with survey distribution and encouraging completion at each center. Topics covered will span center readiness and capacity, training exposure, point-of-care intervention practices with patients, self-efficacy, program acceptability, outcome expectancies, etc. Baseline data will be gathered through the pre-implementation survey, which can be compared to data gathered in the post-implementation survey for program evaluation. The post-implementation survey distribution will occur when all core program components have been delivered and prior to the sustainment/follow-up period.

Most survey items will be face-valid, investigator-generated questions; however, some validated measures will be used. For example, the pre-implementation staff survey will include the Acceptability of Intervention Measure (AIM), Intervention Appropriateness Measure (IAM), the Feasibility of Intervention Measure (FIM) [51], and the Organizational Readiness for Change (ORIC; 12 items) [52]. The post-implementation staff survey will include the Provider REport of Sustainment Scale (PRESS) [53] and the Clinical Sustainability Assessment Tool (CSAT) [54]. Remuneration offered for the completion of each of these surveys, respectively, is $10 in the form of a department store gift card.

*Pre- and post-training tests.* Center staff will attend a training provided by study staff that covers topics like the dangers of tobacco use, harms of environmental exposure to smoke, benefits of tobacco-free workplace policies, impact of tobacco use on patients seen at lung cancer screening programs, and evidence-based methods to deliver cessation interventions and provide tobacco dependence treatment, among other topics. All staff members in attendance will take a brief, face-valid, investigator-generated assessment containing multiple-choice questions covering the topical material immediately preceding and following the training to measure the knowledge gained.

*Program champion reports*. Program champions at each participating center will provide the project team with a monthly report via email. Reports will start during program implementation (to assess changes over time) and continue throughout the 6-month sustainment period. The report will detail the tobacco screenings conducted and smoking cessation interventions patients accepted; other information collected will include patient quit attempts, patient tobacco abstinence, and champion-led tobacco treatment trainings/refreshers, which may be gathered from providers or patient health records by program champions and reported without patient identifiers. Remuneration for the completion of these reports is $100 each, which participating centers can choose to receive via a department store gift card or request via check by invoicing us.

*Group and/or individual interviews.* Qualitative data will include pre-implementation individual interviews with leadership, as well as pre- and post-implementation group and/or individual interviews with staff and (if allowable) patients. Data at pre-implementation will be used to understand centers’ current practices, barriers, and assets to implementation, guide the program adaptation, and solicit feedback on health promotion and provider materials to advance the intervention. At post-implementation, qualitative data will be used to better understand implementation processes, clarify reasons for successes/failures to adopt program components, identify opportunities for improvement, evaluate program penetration and fidelity, and assess potential challenges to sustainment. Group interviews are preferred for qualitative data collection, but individual interviews will be conducted upon center request. Remuneration for participating in a group or individual interview is provided via a department store gift card at $30 per person per interview.

#### 2.5.5. Dissemination Process (Goal 3)

*Other lung cancer screening centers*. The step-by-step implementation guide will be created as the active implementation in enrolled centers draws to a close and will include a description of Project SWITCH and its components for center-led implementation, with or without technical assistance from the study team. The guide will also have sample policies and procedures, frequently asked questions, links to the program website where sample health education and provider materials can be accessed, etc. This guide will be sent to screening programs across Texas and nationally that did not participate in Goal 2, along with an offer to assist lung cancer screening centers through technical assistance, including resource provision or other guidance to facilitate self-led program implementation. This dissemination process, which will begin once the step-by-step implementation guide is finalized, will continue through the end of the grant.

*Community-at-large.* All materials, including training recordings, will be provided for public use on a webpage and will be promoted through study staff’s participation as an exhibitor or attendee in relevant professional meetings, by attending health fairs and other community events, and through quarterly newsletter distribution and continuing education bimonthly blasts. This dissemination process, once started, will continue through the end of the grant.

*Academic dissemination*. Dissemination activities will also include academic dissemination through abstracts, presentations, and manuscript publications. These dissemination activities occur throughout the grant timeline and may span beyond it.

### 2.6. Data Management and Analysis

#### 2.6.1. Qualitative Procedures

*Data Collection.* Our aim guiding data collection will be to explore key stakeholders’ (leadership, staff, and patients) perspectives, experiences, attitudes, and needs around treating tobacco dependence to (1) identify possible barriers and facilitators, (2) enhance adaptation, and (3) understand reasons underlying the implementation of or failure to implement a tobacco-free workplace program within their centers. Criterion sampling, a purposeful sampling strategy that entails selecting participants who meet predetermined criteria of interest, will be used to recruit interview participants [55]. The criteria include leadership and staff from participating centers who will be involved in program implementation in some capacity and patients who are ≥18 years old, English speakers, and current or former people who used tobacco. The program champion/s at the enrolled centers will coordinate selection and scheduling of interview participants in consultation with program team members.

Semi-structured interview guides will be used to conduct virtual group and/or individual interviews lasting ~35–60 min with staff and patients at two time points, pre- and post-implementation, using separate semi-structured interviews. Pre-implementation individual interviews will be conducted with center leaders. All interviews will be conducted by trained qualitative researchers. Study participants will orally consent to and be informed of the nature of the study, that only de-identified data will be retained, and their rights as participants prior to the interview. Qualitative procedures will be recorded with the permission of attendees and transcribed by a third-party, HIPAA-compliant company. The transcripts will be reviewed relative to the recordings, making corrections as necessary and ensuring de-identification prior to destroying the recordings. This process will be done on an ongoing basis; recordings are stored on a password-protected, secure computer server.

The research aims and prior research experience will guide the development of the three different types of interview guides (leadership, employee, and patients, N = 5 in total) that will be tested and adjusted according to responses in the field. As part of the formative evaluation procedures, pre-implementation interview guides will focus on collecting data to facilitate adjusting the program to the needs of the center and the particular populations they serve, e.g., participants’ opinion of printed educational materials to invite cooperation in tailoring materials that accord with individual centers’ populations; current tobacco-free policies, tobacco assessments, cessation services, and tobacco cessation trainings offered; anticipated barriers and facilitators to implementation of a tobacco-free workplace program; and organizational as well as personal attitudes towards implementing such programs. In alignment with the project summative evaluation aims, the post-implementation interview guides will focus on assessing the overall program effectiveness, the extent to which program components were implemented, any program modifications made, implementation barriers and facilitators, and any recommendations for program improvement.

*Data Analysis.* Qualitative data analysis will be conducted immediately following the 2 data collection phases, pre- and post-implementation of the program, which vary according to the continuous cycle of recruiting participating centers. A rapid qualitative analysis approach [56] and matrix analysis [57] will be used to enhance the timely discovery of findings that can better inform intervention development [58] and adaptation to local contexts to further support uptake and understand reasons underlying the extent to which implementation fidelity and other outcomes were met or not. Qualitative data is key within Hybrid Type 3 mixed methods designs to expand or elaborate upon quantitative findings, thus bolstering program evaluation by connecting findings from both qualitative and quantitative datasets [59]. Rapid qualitative analysis was selected as most appropriate for this study, as it was developed to respond to the need for qualitative data in a shorter timeframe to inform program implementation in implementation science and health services research. Analysis will be based on the use of summary templates that will be organized according to key predetermined domains corresponding to each interview question for the three different interview guides. Templates will also include another domain of “additional information” to capture any data not encompassed by existing domains. The use of predetermined concepts will serve to target and focus the analytic process, while inclusion of the “additional information” domain will allow for the development of novel analytic constructs. A rapid analytic approach described by Hamilton [56] will be used to analyze qualitative data. Initially, at least two team members trained in rapid qualitative analysis will use relevant templates to summarize the same five transcripts independently to confirm the applicability of domains and consistency of use across analysts to capture domains. The analytic team will meet to discuss and compare summaries to come to an agreement and/or develop, refine, or create new template domains. Following establishment of consistency of domain application, each transcript will be distributed to team analysts and summarized. Summaries will consist of 2–3-page bulleted points that include exemplary quotes. Summaries will then be transferred to matrices corresponding to each interview type and organized according to individual centers, allowing us to systematically compare the breadth and depth of data for each domain across transcripts and sites and develop key themes [57]. Analyst triangulation will be used to ensure the congruence and credibility of findings.

#### 2.6.2. Quantitative Procedures

*Data Collection.* Quantitative data will be collected through 4 different components. (1) Program records such as center enrollment and completion, provider attendance to trainings, number of technical assistance/consultations provided, number of people and professionals reached through health fairs, presentations, the website, etc. (2) Pre- and post-implementation surveys: online surveys will be administered to participating center leadership and staff. These instruments will assess center readiness and capacity, training exposure, point-of-care intervention practices with patients, and implementation outcomes. (3) Knowledge tests: attendees will complete knowledge tests before and after staff training to evaluate the knowledge gained. (4) Monthly program champion reports: program champions compile de-identified reports on the provider interventions offered, patient acceptance, quit attempts, and tobacco abstinence during implementation and through the 6-month post-implementation follow-up period. Knowledge tests and pre- and post-implementation staff surveys will be administered anonymously to encourage honest responses.

*Data Analysis.* Most quantitative data will be analyzed using descriptive statistics (e.g., means, standard deviations, frequencies, etc.) and pre- to post-difference tests to assess objectives. Quantitative data collected through online surveys will be exported into SAS 9.4 for data management and analysis [60]. Due to their anonymous nature, surveys will not be matched at an individual staff level but will be matched at the lung cancer screening center level. Thus, pre- to post-different comparisons will be conducted using 2-sample tests (*t*-test or Mann–Whitney test for continuous variables or chi-square tests/Fisher’s exact test for binary variables). Models will account for nesting within the screening center and will be adjusted for potential confounders, which in our prior work have included various center characteristics (e.g., staff size and patient volume). Effect sizes will be calculated. Statistical analyses will be performed using SAS 9.4 [60]. The statistical significance of changes over time, when applicable, will be determined based on *p* < 0.05.

#### 2.6.3. Mixed Methods Integration

A convergent parallel mixed methods design will be used, in which quantitative and qualitative data will be analyzed separately and merged at the end of each phase. Mixed methods designs are well-suited for the evaluation of complex interventions; quantitative methods can measure program impact while qualitative data elucidate implementation context, processes, and changes [61]. Qualitative analysts will be blind to quantitative results until the merging of component results during the final stage of analysis at the end of each phase. Various types of integration will be used to mix the quantitative and qualitative data (e.g., creating a joint display table). During formative evaluation, qualitative data will be used to build and adapt intervention features to the local context. During implementation, data types will be compared and connected to make ongoing program adjustments. During program evaluation, data types will be merged so that qualitative data can help explain quantitative outcomes.

## 3. Results

Goal 1 is underway. A training curriculum has been created to educate staff, a recording of which has also been made available on the project website [62]. In partnership with the University of Texas MD Anderson Cancer Centers’ Continuing Professional Education group and at our partner centers’ request, we also launched an online asynchronous training on tobacco dependence, offering continuing medical education credits [63]. Six versions of health education materials were created in English and four in Spanish, covering topics such as the benefits of lung cancer screenings, and are available on our website [64]. Materials were created for providers to remind them to ask patients about tobacco use and provide brief interventions and referrals for further care. The step-by-step implementation guide has been drafted and is in the process of team review.

Two lung cancer screening centers (four physical locations of the intended nine or more targeted) are actively participating in Goal 2 to date. Center 1 formally enlisted in the intervention in March 2024, with Center 2 joining 2 months later. Recruitment for Goal 2 was closed in 2025, with no additional lung cancer centers joining the active implementation of Project SWITCH. Formal program evaluation will begin when all implementation data have been collected, but some information on progress and processes within these centers is provided below for context.

Center 1 has 21 full-time staff and serves about 3000 unique patients in Smith County, Texas, US. It has a single physical location and, according to the Health Resources & Services Administration [65,66], is considered urban. About 60% of Center 1’s patients are women; 40% of patients served are White, 15% Black, 10% multi-racial, and 35% “other” race, with about 45% reporting Hispanic ethnicity. Overall, 86% of Center 1’s staff took the pre-implementation survey and 10 participated in qualitative interviews. Center 1 had a comprehensive tobacco-free policy in place at pre-implementation, which was reviewed for any gaps. There was extant tobacco-free workplace signage, but we provided Center 1 with liquid nails to support hanging more signage in “hot spot” areas for tobacco use that were observed on a site visit. During implementation, Center 1 completed tobacco use assessment and nicotine replacement therapy procedures. Our team provided education to 81% of staff, who exhibited a statistically significant 55.6% gain in knowledge. The program champion completed a 5-day Tobacco Treatment Specialist training. Twenty boxes of nicotine replacement therapy have been sent to Center 1 (value = USD 5764) so far via two orders. Center 1 requested 1000 printed copies of health education materials for patients and intervention reminder cards for providers (value = USD 2486).

Center 2 has eight full-time staff and serves about 110 unique adults in their three physical locations in Taylor and Brown counties, serving 16 additional surrounding counties in Texas, US. All these counties are considered rural. About 52% of Center 2’s patients are women; 88% of patients served are White, 10.5% Black, and 1.5% “other” race, with about 1% reporting Hispanic ethnicity. Overall, 80% of Center 2’s staff took the pre-implementation survey and five participated in qualitative interviews. Center 2 had a comprehensive tobacco-free policy in place at pre-implementation, which was reviewed for any gaps. Center 2 was offered custom tobacco-free workplace signage, but they indicated a preference to create it in-house. During implementation, Center 2 completed tobacco use assessment and nicotine replacement therapy procedures. Our team provided education to 80% of staff, who exhibited a nonsignificant 16.7% gain in knowledge. Overall, five staff members completed a 5-day Tobacco Treatment Specialist training. Nine boxes of nicotine replacement therapy have been sent to Center 2 (value = USD 2551) so far. Center 2 requested 1100 printed copies of health education materials for patients and intervention reminder cards for providers (value = USD 871).

The study team meets regularly for discussion and reviews monthly reports to gauge implementation progress. The funder is provided with quarterly and yearly reports.

## 4. Discussion

The implementation of Project SWITCH in centers with lung cancer screening programs, in conjunction with its dissemination, will increase the integration of evidence-based tobacco cessation care practices into participating centers’ workflow. Improved tobacco cessation care and relapse prevention provision in this setting will enhance health outcomes for patients eligible for lung cancer screening, half of whom are projected to currently smoke and the other half who largely consist of former cigarette smokers [19]. Furthermore, Project SWITCH is expected to address lung cancer screening inequities among Black patients, who are disproportionately more likely to develop and die from lung cancer [17], particularly due to the recently revised USPSTF guidelines that have more than doubled the number of Black adults who are eligible for lung cancer screening [16]. If successful, Project SWITCH will provide a replicable framework for implementing evidence-based tobacco-free workplace programs in this setting and may inform public health policies and guidelines that improve care delivery in lung cancer screening centers to reduce the societal burden of tobacco-related cancers.

To date (a little over 2 years into a 3-year grant contract), Project SWITCH has thus far faced some barriers while achieving some successes. The loss of the embedded collaborator in the original grant application, whose healthcare center’s nine locations in a major metropolitan area of Texas formed the basis of Goal 2 (the number of participating lung cancer screening centers), required an unanticipated recruitment period. Resulting setbacks along the grant timeline and their impact on the budget led to a failure to meet the original recruitment target. That said, the benefits of working with the two different healthcare systems (four locations in total) that enrolled in Project SWITCH allow the opportunity to examine implementation in more diverse settings, including but not limited to those serving rural areas that otherwise would not have been achieved. Implementation successes include a high proportion of staff at both centers participating in the tobacco education programming provided by Project SWITCH, which contributed to improvements in their tobacco-related knowledge (achieving penetration goals). The intervention is also helping both centers improve the prioritization of tobacco cessation care by enhancing the visibility of their tobacco-free workplace policy, integrating tobacco use assessments into their workflow, increasing the availability of cessation services, and improving resource availability for patient health education. The study team’s work on all Project SWITCH goals and objectives will continue through 2026 and potentially beyond if a no-cost extension is requested; final outcomes for the work, inclusive of all lessons learned, will be shared with the field thereafter.

In the original grant application, potential barriers to the successful implementation of Project SWITCH were envisioned and included challenges in the retention of participating centers with lung cancer screening programs, as well as ensuring program fidelity. While there is motivation in this setting to enhance tobacco cessation care provision—especially with practices aligned with COE designation—program champion turnover and/or ongoing challenges related to staffing, resource allocation, and/or the prioritization of tobacco cessation care may limit centers’ buy-in needed for continual engagement with the team. To address these potential challenges, a multifaceted approach has been developed that includes the following: (1) leveraging formative evaluation to build capacity and adapt the comprehensive tobacco-free workplace program to the context of centers with lung cancer screening programs; (2) embedding comprehensive tobacco use assessments and brief behavioral tobacco interventions into centers’ workflow (e.g., 5A’s); and (3) providing resources (e.g., signage, dissemination materials, and nicotine replacement therapy) and hands-on technical assistance throughout program implementation. Additionally, the provision of remuneration for the data collection aspects of Goal 2 can motivate participation. Other barriers may certainly arise over time; we will rely on previous experience, meeting participating centers where they are, ensuring attention to relationship building during implementation, equipping partners with resources to consistently provide tobacco cessation care that is commensurate with evidence-based practices, and work closely with our healthcare center partners in overcoming these challenges.

The implementation timeline for Project SWITCH spans 3 years to provide a phased approach to program implementation and dissemination. In the first half of Year 1, the study team will finalize protocols and secure institutional approval, administer pre-implementation surveys, and complete the formative research process with recruited centers to tailor training and dissemination materials to the unique context of each setting. The second half of Year 1 through the beginning of Year 3 will consist of implementing core components of Project SWITCH, including tobacco-free workplace policy adoption, staff and provider education, and integration of tobacco use assessments and cessation services into centers’ workflow, in addition to evaluating program outcomes and implementation processes. Concurrently, a step-by-step implementation guide will be prepared, and contact information for lung cancer screening centers statewide will be coalesced for the project’s dissemination arm. Year 3 will be dedicated to maintaining project fidelity and sustainability in centers that implemented Project SWITCH through logistical support, refresher trainings, and continuous communication with program champions, while also supporting non-participating centers who were solicited for the dissemination phase in self-led implementation through resource provision and technical assistance. The implementation and dissemination of Project SWITCH together will bolster tobacco care provision within centers with lung cancer screening programs in Texas to reduce cancer disparities caused by elevated tobacco use.

A strength of the present work is its mixed methods design. The project team adopted a convergent parallel mixed methods approach whereby quantitative and qualitative data will be concurrently collected and analyzed separately throughout program implementation and merged in the final analysis. The collection of quantitative data will be used to evaluate program outcomes, whereas qualitative results will be used to understand the implementation processes that contributed to such outcomes. Conducting interviews and focus groups with different stakeholders at each center will provide more varied perspectives, yielding a greater and more comprehensive understanding of implementation processes, and will be coded by multiple qualitative researchers, which is another notable strength of this study. Multiple sources of data (e.g., leadership, staff, and patients) and analysts will facilitate data and investigator triangulation, respectively, to strengthen the reliability and level of detail presented in the qualitative data. This approach also allows for the identification of potential discrepancies between data sources (e.g., what leadership thinks is happening vs. what staff say is happening), providing opportunities to address them through tailored implementation strategies. Lastly, Project SWITCH is being adapted from what has been successfully implemented to improve tobacco care provision in a diverse number of healthcare settings [32,35,40].

This study is not without limitations. Surveys will only be administered to lung cancer screening staff at pre- and post-implementation. Consequently, the extent to which individual programming components contributed to shifts in surveyed items, as well as the sustainability of the program past 6 months post-implementation, will be unknown. Surveys will also be anonymous, which will preclude the examination of individual-level changes in survey measures from pre- to post-implementation. Furthermore, it will not be possible to account for any staff turnover that may affect the success of program implementation. A final limitation is that the results may have limited generalizability to other geographical areas where demographics, policies, and cultural attitudes are significantly different from the centers included in this Texas-based study.

## 5. Conclusions

Improving tobacco cessation care provision in centers with lung cancer screening programs is necessary to reduce the incidence and mortality from tobacco-related lung cancers and improve patients’ overall health outcomes. The results of this study will provide an evidence-based framework for adapting, implementing, and disseminating comprehensive tobacco-free workplace programs in lung cancer screening centers. To that end, this study is expected to address the pervasive use of tobacco products among patients eligible for lung cancer screening and reduce the morbidity and mortality associated with cancer in Texas.

## Figures and Tables

**Table 1:** Overview of Project SWITCH’s implementation objectives, measurements, and desired outcomes.

Implementation Objectives	Measurements	Operationalization (Desired Outcomes)
Penetration	Study team records	≥1 tobacco treatment specialist
		≥1 tobacco treatment training
		≥80% of center personnel educated
	Pre- and post-training tests	≥20% knowledge increase
Fidelity	Study team records	Center retainment
	Post-implementation surveys	Delivery of behavioral tobacco interventions to all people who currently/previously smoked
Sustainment *	Program champion report	≥1 new staff completes the pre-recorded training
		>0 monthly NRT distribution
		Consistent delivery of behavioral tobacco interventions to all people who currently/previously smoked

Note. * Desired outcomes measured during the 6-month sustainment period. NRT = nicotine replacement therapy.

**Table 2:** Project SWITCH’s implementation process components and objectives.

Implementation Process Components ^∇^		Process Objectives
E	Conduct pre-implementation quantitative assessments of stakeholders (leadership, providers, other staff)	Establish a baseline for enrolled centers regarding training, knowledge, interventions, implementation concerns, etc., and patient demographics and smoking status
E	Conduct pre-implementation qualitative assessments of stakeholders (leadership, staff, patients)	Understand centers’ current practices, barriers, and assets to implementation; guide the adaptation of culturally informed interventions within centers
E	Create implementation plan/vision for center-specific rollout timeline	Negotiate and set an implementation timeline with concrete steps for completion
P (E)	Review Memorandum of Understanding with center leadership	Achieve mutual understanding and buy-in for process, timeline, and expectations
P	Select program “champions” (>1 person per clinic) with center leadership	Secure cooperation for implementation and assessment procedure execution
P (S)	Ensure sufficiency of tobacco-free workplace policy and enforcement	Establish/verify/refresh an enforceable tobacco-free workplace policy and publicize it broadly
P (I)	Conduct visits (and provide technical assistance) throughout implementation	Build and nurture partnership via presence/contact; achieve technical problem-solving
I (S)	Sponsor center champions to become Tobacco Treatment Specialists	Embed specialized knowledge into the center; equip champions to lead future in-center training provision
I (S)	Champions (with or observed by study staff) provide education about tobacco treatment to center providers	Increase knowledge about how to provide smoking cessation and relapse prevention interventions on site; train-the-trainer for center-led trainings
I	Study staff provide specialized training with intervention roleplay to champions and providers (collaborative learning)	Convey and facilitate replicable smoking cessation behavioral treatment skills with culturally- and trauma-informed approaches
I	Study staff check in regularly with program champions to discuss the implementation process and advise on challenges that arise	Understand implementation processes and opportunities for improvement; advance intervention fit
I (S)	Change systems to facilitate regular and sufficient treatment for smoking	Integrate processes into systems for conducting and documenting regular intervention provision
I (S)	Design and provide passive dissemination materials for patients and providers	Enhance stakeholders’ expectations for assistance and resource knowledge; ease implementation
I (S)	Design, supply, and install tobacco-free signage with consultation of center	Support the messaging of a tobacco-free workplace; empower community policy enforcement
I	Supply a “starter kit” of NRT with dispensation record planning	Make evidence-based medications for cessation available to staff and patients to decrease barriers to care and enhance cessation
I	Conduct post-implementation quantitative assessments of stakeholders	Gather data on implementation to evaluate program penetration, fidelity, sustainment, etc.
I	Conduct post-implementation qualitative assessments of stakeholders	Gather data on implementation to evaluate program penetration, fidelity, sustainment, etc.
S	Work with leadership to budget/obtain funding for additional NRT	Sustain availability and ease of access to medications to address tobacco use
S	Provide continuing education information regularly (e.g., newsletters, weekly email)	Equip providers to sustain/reinforce knowledge through free CEU/CME-associated trainings
S	Prepare center with implementation feedback and suggestions to sustain and enhance policy/practice gains	Provide pre- to post-implementation evaluation results; help equip for practice facilitation through attention to quality improvement opportunities
S	Provide continued support to partner centers	Ensure availability of all reference materials on website; provide technical assistance as needed

Note. ^∇^ Includes implementation strategies, evaluation processes, and resources dispensed, delineated by letter according to the EPIS framework, where E = Exploration, P = Preparation, I = Implementation, and S = Sustainment; NRT = nicotine replacement therapy; CEU = continuing education unit; CME = continuing medical education.

## Data Availability

The study materials and data presented in this study are available upon request from the corresponding author at the email address provided in the byline. The data are not publicly available because the study is ongoing. You can read more about Project SWITCH here: https://www.takingtexastobaccofree.com/lung-cancer-screening-centers (accessed on 30 January 2025), and access various resources here: https://www.takingtexastobaccofree.com/lung-cancer-screening-resources (accessed on 30 January 2025).
